# Silver Ion Implantation into Orthodontic Stainless-Steel Materials: Physicochemical Characterization and In Vitro Microbiological Evaluation

**DOI:** 10.3390/jfb17100487

**Published:** 2026-09-27

**Authors:** Berta Furió-Alonso, María del Carmen De Lama-Odría, Lucía Pallarés, Danica Nikolic-Jovanovic, Javier Gil, Andreu Puigdollers

**Affiliations:** 1 Department of Orthodontics, School of Dentistry, Universitat Internacional de Catalunya, C/Josep Trueta s/n, 08195 Sant Cugat del Vallés, Spain; bertafurioa@uic.es (B.F.-A.);; 2 Bioinspired Oral Biomaterials and Interfaces (BOBI), Department of Materials Science and Engineering and Barcelona Research Centre in Multiscale Science and Engineering (CCEM), Barcelona East School of Engineering (EEBE), Universitat Politècnica de Catalunya—BarcelonaTech (UPC), Av. Eduard Maristany, 16, 08019 Barcelona, Spain; maria.de.lama@upc.edu; 3 Bioengineering Institute of Technology, Universitat Internacional de Catalunya, C/Josep Trueta s/n, 08195 Sant Cugat del Vallés, Spain; l.pallaresferrer@gmail.com

**Keywords:** silver implantation, orthodontic brackets, stainless-steel, dental caries, tooth demineralization, dental plaque, antibacterial, surface hardness, friction coefficient

## Abstract

Fixed orthodontic appliances create retention sites for bacterial biofilm, increasing the risk of white spot lesions and periodontal complications. Silver ion (Ag) implantation offers a potential solution by conferring antimicrobial properties while preserving mechanical integrity. This study evaluated the physicochemical properties, Ag release, biocompatibility, and antibacterial efficacy of AISI 301 stainless-steel orthodontic brackets (Ormco Bios) modified by silver ion implantation using a Pulsed Filtered Cathodic Arc Vacuum System. Physical and micro-structural surface characterizations—including SEM/EDS, interferometric roughness analysis, contact angle measurements, nanoindentation hardness testing, and friction coefficient determination (under dry and saliva-lubricated conditions)—were conducted directly on the modified brackets. In compliance with international testing standards requiring defined geometric areas, AISI 301 flat samples of same chemical composition and microstructure were used to quantify Ag release in artificial saliva (cumulative silver ion release in artificial saliva was quantified via ICP-MS over time, with measured concentrations corrected for the volume extracted at each sampling interval), as well as to perform in vitro biological (human fibroblast cytocompatibility) and microbiological assays against seven bacterial strains (*Streptococcus mutans*, *Streptococcus gordonii*, *Lactobacillus salivarius*, *Lactobacillus acidophilus*, *Porphyromonas gingivalis*, *Enterococcus faecalis*, and *Staphylococcus aureus*). SEM/EDS confirmed successful Ag incorporation (0.93 wt.%) on the brackets. Surface roughness remained unchanged (0.16–0.17 mm), whereas hydrophilicity, surface energy, and nano-hardness increased significantly. Friction coefficients decreased under both dry and saliva-lubricated conditions. ICP-MS analysis revealed Ag release stabilizing at 119 ± 5.0 ppb after one week. Biocompatibility testing demonstrated no cytotoxic effects on fibroblasts. Furthermore, modified surfaces exhibited marked antibacterial activity against *Lactobacillus* spp., *P. gingivalis*, *E. faecalis*, and *S. aureus*, though no significant inhibition was observed against *S. mutans* or *S. gordonii*. Ag ion implantation directly improves the mechanical and tribological properties of stainless-steel orthodontic brackets. The use of standardized specimens confirmed steady Ag release, cytocompatibility, and selective antibacterial efficacy, supporting Ag implantation as a viable surface modification strategy for clinical orthodontic applications.

## 1. Introduction

The placement of orthodontic appliances, both fixed and removable, introduces substantial modifications to the oral ecosystem that compromise the patient’s ability to maintain adequate oral hygiene [1,2]. Fixed orthodontic appliances, such as brackets, arch-wires, elastomeric ligatures, bands, miniscrews and other components bonded to dental surfaces, create multiple retention sites for bacterial biofilm that significantly complicate oral hygiene procedures for patients [3,4]. These devices limit the patient’s capacity to effectively clean their teeth through conventional brushing and flossing techniques, while simultaneously reducing the natural self-cleansing effect of saliva and the tongue on dental surfaces [5].

In addition, dental plaque accumulation around orthodontic appliances follows a distinctive pattern characterized by temporal quantitative and qualitative changes in the microbial composition [6]. During the first weeks following fixed appliance placement, species such as *Streptococcus mutans*, *Lactobacillus spp*., and *Actinomyces spp*. predominate; and these acid-producing bacteria are responsible for the development of white spot lesions [3,7]. These cariogenic bacteria exhibit a particular affinity for bracket surfaces, especially in the presence of sucrose, which significantly enhances their adherence and biofilm formation capacity [8,9]. Additionally, periodontal pathogens such as *Porphyromonas gingivalis*, *Aggregatibacter actinomycetemcomitans*, *Tannerella forsythia*, and *Treponema denticola* progressively colonize orthodontic appliances, contributing to gingival inflammation and periodontal complications [2,10]. *Enterococcus faecalis* was included as a representative, highly resilient Gram-positive opportunistic pathogen to test the coating against resistant biofilm-forming species Although commonly linked to endodontic infections, *E. faecalis* is frequently isolated from the oral cavity of patients with fixed orthodontic appliances, where plaque retention sites favor opportunistic colonization. Within two to three weeks following bonding of the orthodontic appliances, mature plaque is observed in these areas, while plaque on the gingival enamel surface remains in early developmental stages.

Biofilm formation on orthodontic appliances constitutes a complex process that initiates with the adhesion of early colonizers such as *Streptococcus salivarius* and *Streptococcus oralis* to bracket surfaces, followed by the incorporation of late colonizers that establish a mature three-dimensional structure [11]. This mature biofilm, composed of bacteria embedded in an extracellular matrix, produces organic acids that induce dental enamel demineralization, a process that constitutes the initial event in caries development [5]. Orthodontic appliances create a critical bacteria-material interface where bacterial biofilm establishes and proliferates, driving interest in material-based solutions that could function independently of patient behavior [12]. The increase in caries-associated bacterial counts in biofilm facilitated by the retentive surfaces of the orthodontic materials and the reduction in salivary pH result in enamel demineralization, a process that is significantly aggravated in the presence of inadequate oral hygiene. As a consequence, initial enamel caries leads to subsurface demineralization beneath the outer enamel layer, whose light reflection differs from that of surrounding healthy enamel, resulting in a white, chalky appearance known as white spot lesions (WSLs) [13]. These lesions manifest as small lines around brackets or as more extensive demineralized areas, with or without cavities, and represent a frequent complication of modern orthodontic treatment. In addition to WSLs, biofilm formation on orthodontic appliances induce significant changes in the microbial profile due to a qualitative shift from aerobic to anaerobic microflora, increasing the risk of generalized gingival inflammation and periodontal disease [14].

The existing scientific evidence demonstrates that the orthodontic treatment exerts a negative impact on oral hygiene, making the creation and implementation of adequate oral hygiene protocols and effective preventive strategies a research and clinical priority. Conventional preventive strategies, such as oral hygiene protocols, fluoride applications, and antimicrobial rinses, present significant limitations due to their dependence on patient’s compliance, and only provide a temporary protection as a rapid bacterial recolonization occurs after their cessation [3,15].

In response to these limitations, contemporary research has focused on the development of material-based solutions, that works independently of patient’s behavior [16]. So far, multiple strategies have been investigated to prevent the biofilm formation on orthodontic brackets, including coatings with metallic nanoparticles (silver, zinc oxide, and titanium dioxide), polymeric coatings (polyethylene glycol and chitosan), and surface modifications through plasma treatment [5,7,12]. Through these approaches, antibacterial properties can be achieved by either contact killing, ion release that triggers bacterial death, surface chemical modification that convey an antifouling effect or photocatalytic bacterial inactivation [17].

Among surface modification techniques, physical vapor deposition (PVD), electrochemical deposition, and plasma immersion ion implantation and deposition (PIII&D) have been investigated to confer antimicrobial properties to metallic devices. However, conventional deposition methods produce a discrete coating layer with a sharp interface, often requiring intermediate adhesion layers (e.g., chromium or titanium buffer layers) to prevent delamination. In orthodontic brackets subjected to high mechanical friction and stress, such coatings risk peeling, mechanical failure, or dimensional alteration of the bracket slot. In contrast, Pulsed Filtered Cathodic Arc (PFCA) ion implantation bombards high-energy Ag ions directly into the atomic sub-surface of the stainless-steel substrate without generating a physical interface. Furthermore, the 90° magnetic filter eliminates macroparticles typical of unfiltered cathodic arcs, preserving the original nanoscale surface roughness and critical slot geometry while enhancing tribological performance [18,19,20,21].

In this context, the present study investigated the potential of silver ion (Ag) implantation as a surface modification of stainless-steel orthodontic brackets via Pulsed Filtered Cathodic Arc evaporation deposition (PFCA) to enhance their antibacterial performance without compromising the physicochemical or mechanical properties of the material. To fully elucidate the effects of Ag implantation by the PFCA approach, the surface morphology, roughness, wettability, hardness, frictional behavior, Ag release, biocompatibility, and antibacterial assays were performed.

The null hypotheses tested in this study were: 1. Silver ion implantation onto stainless-steel orthodontic brackets does not exert any antibacterial or bactericidal activity against tested oral microorganisms. 2. The silver ion implantation process negatively alters the tribological interaction between the arch wire and the bracket slot by increasing friction, thereby impairing orthodontic sliding mechanics.

## 2. Materials and Methods

### 2.1. Samples

The study was carried out with 99 stainless-steel flat samples with initial dimensions of 10 × 10 × 1.5 mm. The chemical composition was measured by means of dispersing energy of X-ray (EDS) and corresponded to AISI 301: Fe (balance), Cr (16.4 ± 0.7%), Ni (8.0 ± 0.6%), Mn (2.2 ± 0.2%), Si (0.8 ± 0.2%), and C (0.010 ± 0.007%). Stainless steel is the same material used to machine the brackets used in this study. For the friction coefficient assays, the studies were carried out with Bios stainless-steel brackets, BIOS Brxaket0.018Upper3Left7T10A0DO (Ormco Corporation, Brea, CA, USA). The characteristics were: slot size of 0.457 mm, corresponding to the upper arch and the left side with 7° of torque and angulation of 10° and 0° of rotation. A total of 195 brackets with serial numbers 0089765447 batch were used, and the chemical analyses performed were virtually identical to those of the brackets: Fe (balance), Cr (16.2 ± 0.7%), Ni (8.0 ± 0.5%), Mn (2.0 ± 0.3%), Si (0.7 ± 0.1%), and C (0.011 ± 0.005%). Bracket friction was achieved using Ormco stainless-steel (AISI 301) arch wires (Alexander WiresSS 0.017 × 0.025 size 3 Upper) with the following rectangular dimensions: 0.432 × 0.635 mm (orthodontic arch wires) from Ormco Corporation (Brea, CA, USA). Sixty-five arch wires were used for the tests (Series number 038766547-038766555). The composition of the arch wire material reported by the manufacturer was confirmed by means of disperse energy of X-ray (EDS), being comprised by Fe (balance), Cr (16.7 ± 0.8%), Ni (7.9 ± 0.7%), Mn (2.0 ± 0.3%), Si (0.8 ± 0.2%), and C (0.011 ± 0.009%). The chemical composition was determined for 10 flat samples, 10 brackets and 10 arch wires.

All tests (physicochemical characterization, ion release, tribology, cytocompatibility, and microbiology) were conducted on independent, brand-new specimens. No brackets, flat specimens, or arch wires were reused across different experiments to avoid any risk of surface contamination or altered topography/tribology.

Number and type of samples:

- Chemical composition: 10 flat samples + 10 brackets +10 arch wires.
- SEM observations: 5 flat samples (2 control and 3 silver-implanted) +5 brackets (2 control and 3 silver-implanted).
- Roughness: 5 brackets × 2 treatments (control and silver-implanted) + 5 arch wires (only control) = 10 brackets + 5 arch wires.
- Contact angle and surface energy: 10 brackets × 2 treatments (control and silver-implanted) × 3 solutions = 60 brackets.
- Ion release: 5 brackets × 2 treatments (control and silver-implanted) × 5 times = 50 brackets.
- Nanoindentation: 10 brackets × 2 treatments (control and silver-implanted) + 10 arch wires = 20 brackets + 10 arch wires.
- Friction tests. 10 brackets × 2 treatments (control and silver-implanted) × 2 conditions (dry and saliva) + 20 arch wires × 2 conditions (dry and saliva) = 40 brackets and 40 arch wires.
- Biological tests: 2 flat samples × 2 growth media (control and silver-implanted) = 4 flat samples.
- Protein adsorption: 6 flat samples × 2 treatments (control and silver-implanted) × 2 types of proteins = 24 flat samples.
- Microbiological tests: 4 flat samples × 7 strain bacteria × 2 treatments (control and silver-implanted) = 56 flat samples.

TOTAL: 99 flat samples + 195 brackets + 65 arch wires.

### 2.2. Ion Implantation Technique

Prior to ion implantation, all samples (AISI 301 stainless-steel 45 flat samples and 93 brackets) underwent a standardized ultrasonic cleaning protocol consisting of sequential 5 min cycles in distilled water, ethanol, and acetone, with each cycle repeated three times. Samples were subsequently dried under a continuous flow of nitrogen gas and stored in a desiccator under controlled conditions until processing.

Silver ion (Ag) implantation was performed using a Pulsed Filtered Cathodic Arc Source system (PFCA-450, Beijing, China) equipped with a 90° curved magnetic filter to eliminate metallic macroparticles (droplets). The vacuum chamber was initially evacuated to a base pressure below 5 × 10^−3^ Pa. High-purity silver cathodes (99.99% pure) were evaporated via pulsed cathodic arc discharge.

The operational implantation parameters were set as follows: an accelerating negative pulse bias voltage applied to the target/substrate of −40 kV, a pulse frequency of 20 Hz, a pulse duration of 200 µs, and an arc current of 70 A. The total implantation process was carried out for 200 min to achieve a nominal target silver fluence (dose) of 4 × 10^17^ ions/cm^2^. The substrate temperature was maintained below 70 °C throughout the treatment to prevent thermal distortion or microstructural alterations of the stainless steel. The modified samples were designated as SS-Ag, while untreated samples served as the control group (SS).

### 2.3. Physicochemical Characterization

#### 2.3.1. Scanning Electron Microscopy (SEM) and Energy Dispersive X-Ray Spectrometry (EDS)

Surfaces were examined using a JEOL 6400 scanning electron microscope (JEOL, Tokyo, Japan) with a resolution of 15 nm and an acceleration voltage of 20 kV. Gold sputter-coating was not required as the samples were sufficiently electrically conductive. The microscope was coupled with an energy-dispersive-ray spectroscopy (EDS) microanalysis system (Oxford Instruments, Oxford, UK). The samples observed were 5 brackets and 5 flat samples (2 untreated and 3 ion-implanted for each one). For EDS, 10 flat samples, 10 brackets, and 10 arch wires were analyzed.

#### 2.3.2. Roughness

Surface roughness was evaluated using a white light interferometric microscope (Wyko NT1100, Veeco, Plainview, NY, USA). To establish the baseline surface topography relevant to friction behavior, biocompatibility, and microbiological adhesion, measurements were performed over an analysis area of 459.9 × 604.4 mm^2^. For the bracket samples were studied 10 brackets (5 control and 5 silver-implanted) and 5 arch wires, five distinct locations were analyzed across five untreated control brackets (*n* = 25 measurements) and five silver-implanted brackets (*n* = 25 measurements). In addition, ten as-received orthodontic arch wires were analyzed (five measurements per arch wire, *n* = 25 measurements) to characterize the countersurface involved in sliding mechanics. Data processing and topological analysis were conducted using Wyko Vision 32™ software (Veeco, USA). A Gaussian filter with a cut-off value of λc = 0.8 mm was applied to separate waviness and form error from the surface roughness profile. Measurements were performed on three different regions of each sample type to determine the roughness parameters *Sa*, defined as the arithmetic mean roughness, and *Sm* that represents the mean spacing between successive peaks. Additionally, the area index parameter, defined as the ratio of the real surface area to the nominal surface area, was calculated from the total surface.

#### 2.3.3. Contact Angle Measurements and Surface Free Energy (SFE) Determination

The wettability and SFE of the samples were assessed by the static sessile drop method. Contact angle measurements were performed using an OCA15 Plus optical tensiometer (DataPhysics Instruments GmbH, Filderstadt, Germany) equipped with SCA20 software (version 2.1) for image acquisition and data analysis. Three probe liquids with well-characterized surface tension components were used: water (polar), diiodomethane (non-polar), and formamide (polar). These analyses were carried out on the surface of the 60 brackets (10 brackets untreated and 10 brackets with silver implanted for the three different liquids). A fixed droplet volume of 2 µL was dispensed onto each sample surface at a controlled dosing rate of 1 µL/s. Four independent measurements per probe liquid (*n* = 4) were performed on each sample type to ensure reproducibility. The total SFE of the solid surface ($\gamma_{S}$), together with its dispersive ($\gamma_{S}^{d}$) and polar ($\gamma_{S}^{p}$) components, was calculated using the Owens–Wendt–Rabel–Kaelble (OWRK) method. This approach is based on the Young–Dupré equation, which relates the measured contact angle (θ) to the surface tension components of both liquid and the solid phases. For this purpose, the surface tension values for water, diiodomethane, and formamide were used to calculate the SFE values in Equations (1) and (2):

(1) $$\gamma_{S}=\gamma_{SL}+\gamma_{L}\cos\theta$$

(2) γL(1+cosθ)=2((γLdγSd)12+(γLpγSp)12)

where $\gamma_{S}$ is the surface free energy of the solid; $\gamma_{L}$ is the surface tension of the probe liquid; ($\gamma_{SL}$) is the interfacial free energy between the liquid (L) and the solid (S); θ is the static contact angle between (L) and (S); and $\gamma^{d}$ and $\gamma^{p}$ denote the dispersive and polar components of the respective surface tension, for both (S) and (L) phases.

By measuring contact angles with the three probe liquids of known $\gamma_{L}^{d}$ and $\gamma_{L}^{p}$, the unknown $\gamma_{S}^{d}$ and $\gamma_{S}^{p}$ were resolved by linear regression, and the total SFE ($\gamma_{S})$ was obtained as their sum (Equation (3)).

(3) $$\gamma_{S}=\gamma_{S}^{d}+\gamma_{S}^{p}$$

#### 2.3.4. Ion Release

The brackets were placed individually in 15 mL Falcon tubes containing artificial saliva and incubated at 37 °C with agitation (220 rpm) until sample collection, which took place on days 1, 3, 7, 14, and 21. At those times, a 1 mL volume of artificial saliva was withdrawn from each tube, and the collected samples were stored at 4 °C until final analysis was performed using inductively coupled plasma mass spectrometry (ICP-MS). Thus, 5 brackets were used at each of the 5 time points, for a total of 25 brackets used without treatment and another 25 brackets with silver ion implantation. For this purpose, the collected samples were thawed and transferred to 16 mL plastic tubes for ICP-MS (polypropylene) that had been previously cleaned with 2% nitric acid. The concentration of silver ions released at each time point was quantified using an Agilent Technologies 7800 ICP-MS system (Agilent Technologies, Santa Clara, CA, USA). The ISO 10993-12 standard was followed [22]. The chemical composition of the artificial saliva is shown in Table 1 [23,24,25].

#### 2.3.5. Nano-Hardness Test

Surface nano-hardness of the untreated (control) and silver ion-implanted brackets and original arch wires were evaluated using a depth-sensing nanoindentation system (NanoIndenter XP MTS, Minneapolis, MN, USA) equipped with a calibrated Berkovich diamond indenter tip (tip radius 20 nm). The loading conditions were a constant strain rate of 0.05 s^−1^ until reaching maximum load and a dwell time 10 s at peak load to allow for thermal drift correction and to dissipate creep deformation. To isolate the mechanical response of the thin Ag-implanted surface layer (3–200 nm thick) and prevent substrate compliance effects, tests were performed under the continuous stiffness measurement (CSM) mode. This mode continuously records hardness and elastic modulus as a function of penetration depth. To satisfy the standard 10% rule (ISO 14577) [23], wherein the maximum indentation depth must not exceed 10% of the modified layer thickness, load-controlled indentations were executed with a maximum peak load of 1.5 mN (or depth limit set to 15 nm). A total of 20 brackets (ten for untreated and 10 silver-implanted) and 10 arch wires were used and three nanoindentations (spaced 20 mm apart to avoid an overlap of plastic deformation zones) were conducted per sample group.

#### 2.3.6. Friction Coefficients

Static and dynamic friction coefficients were measured under dry conditions for 40 stainless-steel arch wires tested against orthodontic brackets (*n =* 20 for the SS group and *n* = 20 for SS-Ag). Ten brackets of each one were tested in dry conditions and with artificial saliva. The arch wires were not treated. Friction force values were determined under two conditions: dry and immersed in artificial saliva at 37 °C (Figure 1). The assays were performed using an MTS Bionix universal testing machine (MTS Systems Corporation, Minneapolis, MN, USA) at a crosshead displacement rate of 0.1 mm/min [26], selected to closely simulate the sliding mechanics of an installed arch wore-bracket assembly under normal oral conditions. The tests were conducted using treated and untreated brackets with original orthodontic wires. Wire lengths of 100 mm without angulation were used and were tied to the brackets with elastic ligatures. The static coefficients of friction were obtained from the force at which the movement between the bracket and the orthodontic arch wire began. Subsequently, 10 cycles of movement between the bracket and the wire were performed within a 20 mm range, and the average value of the dynamic coefficient of friction was determined.

Dry and saliva (Figure 1) friction tests were performed on brand-new bracket-wire systems; they were not reused, as this could alter the surface topography.

### 2.4. Biological Characterization

#### 2.4.1. Cell Viability Assays

Primary human gingival fibroblasts cells (ATCC PCS-201-018), hereafter HGF (passage 6), and human foreskin fibroblasts (ATCC SCRC-1041), assigned as HFF-1 (passage 21), were obtained from the American Type Culture Collection (ATCC, Manassas, VA, USA). HGF cells were cultured in low-serum Fibroblast Growth Medium (FGM) supplemented with 5 ng/mL recombinant human bFGF, 2 mM L-glutamine, 50 µg/mL ascorbic acid, 1 µg/mL hydrocortisone, 5 µg/mL recombinant human insulin, 2% fetal bovine serum (FBS), and 1% penicillin/streptomycin (P/S). HFF-1 cells were cultured in Dulbecco’s Modified Eagle Medium (DMEM) supplemented with 15% FBS and 2 mM L-glutamine. All cells were maintained at 37 °C in a humidified atmosphere containing 5% CO_2_.

For the preparation of extracts for indirect cytotoxicity assays, flat specimens (*n* = 2 per group) of untreated (SS) and silver-implanted (SS-Ag) groups were immersed in complete supplemented DMEM medium (containing 15% FBS and 2 mM L-glutamine for HFF-1 cells), while two additional specimens per group were immersed in complete supplemented FGM medium (containing 5 ng/mL recombinant human bFGF, 2 mM L-glutamine, 50 µg/mL ascorbic acid, 1 µg/mL hydrocortisone, 5 µg/mL recombinant human insulin, 2% FBS, and 1% P/S for HGF cells). In compliance with ISO 10993-5:2009 and ISO 10993-12 guidelines [22], each specimen (total surface area of 2.6 cm^2^) was immersed in 2.08 mL of the corresponding complete growth medium to achieve a standardized surface area-to-extraction volume ratio of 1.25 cm^2^/mL. Incubation was carried out under static conditions at 37 °C in a 5% CO_2_ humidified atmosphere. Extracts were collected at intervals of 1, 3, 7, 14, and 21 days, stored at −20 °C, and filtered through a 0.22 μm syringe filter immediately prior to cell exposure

For cytotoxicity testing, cells at 80% confluency were harvested by trypsinization and seeded at a density of 1 × 10^4^ cells/well in 96-well plates using their corresponding complete growth media. The final passage considered for the assay were passage 6 for HGF and passage 21 for HFF-1 and they were used to perform the viability assays in triplicates. After 24 h of adhesion, the growth medium was replaced with 200 µL of extraction medium collected at days 1, 3, 7, 14 and 21 from both SS and SS-Ag samples. Cells were then incubated for an additional 24 h, after which cell viability was assessed using a resazurin reduction assay. Briefly, cells were incubated for 4 h with a 15 µg/mL resazurin solution prepared in the corresponding culture medium (FGM or DMEM). Fluorescence was measured at *λ_ex_* = 560 nm/*λ_em_* = 590 nm, and cell viability was expressed as a percentage relative to the untreated control.

#### 2.4.2. Protein Adhesion Assays (Acquired Pellicle Simulation)

For these assays, flat samples were considered. Prior to protein adsorption, all samples were cleaned for 2 h with 70% ethanol under agitation (250 rpm), washed three times with PBS 1X and sterilized under UV light inside a BSL 2 cabinet (15 min each side of the platelets).

Protein adsorption was evaluated using artificial saliva containing either type I-S bovine submaxillary gland mucin (Sigma-Aldrich, M3895, St. Louis, MO, USA) or human salivary α-amylase type XIII-A (Sigma-Aldrich, A1031-1KU, St. Louis, MO, USA) at concentrations of 3.1 mg·mL^−1^ and 539.5 μg·mL^−1^ (65 U·mL^−1^), respectively. Even though the evaluation of independent adsorption of mucin and amylase does not reproduce the complexity of the formation of the acquired pellicle formation, it does provide an understanding of the effect of silver ion implantation on the adsorption of the main two protein components of this pellicle. SS and SS-Ag samples (6 per group × 2 (control and silver-implanted) × 2 proteins = 24 samples) were incubated with 1 mL of the corresponding protein solutions at 37 °C under agitation (250 rpm) for 24 h. Regarding the time of incubation, although the maximum maturation of in vivo salivary pellicle has been reported after 2 h, the immersion time in this study was extended to 24 h to correlate with the first microbial attachment and accumulation on dental materials [27]. After this time, three samples per group were used for the quantification of adhered protein while the remaining samples were used for evaluating the changes in the adhered protein on the surface wettability and roughness. For protein quantification, the samples were washed twice with sterile PBS, and the adsorbed proteins were desorbed overnight using 500 µL of 2% (*w*/*v*) sodium dodecyl sulphate (SDS) in PBS. Protein concentration in the SDS extract was determined using a bicinchoninic acid (BCA) assay according to manufacturer instructions. Standard curves were prepared for mucin and α-amylase. Absorbance was measured at 562 nm, and the amount of adsorbed protein was calculated form the corresponding standard curve and expressed as the percentage of the total protein initially applied to the sample surface. Regarding the analysis of the effect of the adhered protein layer on surface wettability and roughness, white light interferometric microscopy and static water contact angle determination were performed, correspondingly, following the methods explained in Section 2.3.2 and Section 2.3.3.

#### 2.4.3. Microbiology Assays

a. Bacteria strains and growth conditions

*Streptococcus mutans* (ATCC 25175), *Streptococcus gordonii* (ATCC 10558), *Enterococcus faecalis* (ATCC 29212), *Porphyromonas gingivalis* (ATCC 33277), *Streptococcus sanguinis* (ATCC10556), *Lactobacillus salivarius* (ATCC 10942) and *Lactobacillus* spp. (ATCC 14435) were recovered from cryopreserved stocks (−80 °C). *S. mutans* and *S. gordonii* were cultured aerobically in Tryptic Soy Broth (TSB; PanReac AppliChem Spain) and Tryptic Soy Agar (TSA). *E. faecalis* was cultured under aerobic conditions in Brain Heart Infusion (BHI) broth and agar (Biorad, Hercules, CA, USA) while *Lactobacillus* spp. was cultured in Man, Rogosa and Sharpe (MRS) broth and agar (Merck, Darmstadt, Germany) *P. gingivalis* was grown under anaerobic conditions using the BD GasPakTM anaerobic system (BD Diagnostics, Sparks, MD, USA) in BHI medium (Biorad, Hercules, CA, USA) supplemented with 5% (*v*/*v*) defibrinated sheep blood, 5 µg/mL hemin and 1 µg/mL vitamin K1. In all cases, bacteria grew at 37 °C.

b. Biofilm formation and determination of changes in bacteria count by colony forming unit (CFU) and resazurin metabolic assays.

Bacterial suspensions were standardized to an optical density (OD) of 0.8 (equivalent to a 0.5 McFarland standard). The total number of samples were: 4 samples × 2 treatments (SS and SS-Ag) × 7 bacteria strains = 56 samples. The inoculated samples were then incubated for 24 h at 37 °C under the optimal culture conditions previously described for each bacterial strain. Briefly, 1 mL of the standardized bacterial suspension was used to cover each sample. In parallel, four wells containing 1 mL of the same bacterial inoculum without a sample served as positive growth control, whereas four wells containing 1 mL of sterile culture broth served as negative controls. In the case of *S. gordonii* and *S. mutans*, TSB broth was supplemented with 1% (*w*/*v*) or 5% (*w*/*v*) sucrose to promote biofilm formation or the growth of bacteria under cariogenic conditions, correspondingly [28,29]. Following biofilm formation, SS and SS-Ag samples were transferred to sterile 24-well plates and were gently washed with PBS 1X to remove non-adherent bacteria. A resazurin assay was performed immediately by incubating each sample for 30 min at 37 °C in the dark with 1 mL of a resazurin solution prepared in PBS 1X (5 µg/mL). The resazurin incubation time was limited to 30 min as bacteria metabolize more rapidly in this reagent than mammalian cells. Therefore, a shorter exposure time guarantees a linear signal and prevents over-reduction. The positive growth controls were also incubated in parallel with the same resazurin solution to later determine the percentage of bacteria growth on the stainless-steel samples. In addition, blank wells containing only PBS/resazurin were included for background correction. Subsequently, 150 µL aliquots were transferred to black 96-well plates, and fluorescence intensity determine using an Infinite M200 PRO multimode microplate reader (Tecan Group Ltd., Männedorf, Switzerland).

Following the metabolic activity assays, samples were transferred to sterile tubes containing 1 mL of PBS 1X and adherent bacteria were detached by applying 4 cycles of sonication (3 min)/vortexing (5 min) following previous protocol optimization by the research group to ensure full biofilm detachment. The resulting bacterial suspensions serially diluted (10-fold dilutions) in PBS and appropriate dilutions were plated onto the corresponding agar medium for each bacterial strain. Plates were incubated following the growth conditions indicated on Section 2.4.3. a and the colonies were later counted. Bacterial viability was expressed as the logarithm (log10) of CFU per milliliter.

### 2.5. Statistical Analysis

All statistical analyses were performed using Stata 14 software (StataCorp LLC, College Station, TX, USA) (version 3.2) or OriginPro 2020 (64 bit) (OriginLab Corporation, Northampton, MA, USA) softwares. Shapiro–Wilk and Levene’s tests were performed to determine data normality and variance homogeneity. The significance level for all tests was set at α  =  0.05. Differences between groups were assessed using Student’s *t*-test, one-way analysis of variance (ANOVA), and Tukey’s post hoc multiple comparisons test. A significance level of *p* < 0.05 was applied as the criterion for statistical significance.

## 3. Results

### 3.1. Surface Characterization and Ag Ion Release Analysis

The morphological features of the SS and SS-Ag samples were analyzed using SEM. SEM images showed that Ag deposition did not result in a significant modification of the surface morphology (Figure 2). The EDS analysis demonstrated a homogenous deposition of Ag ions with a quantitative estimation of the ion content of 0.93 ± 0.60 weight percentage (Table 2). SEM observations were confirmed via optical interferometry analysis (Figure 2C,D.1) as the average *Sa* and *Sm* values for the SS and SS-Ag were 0.16 ± 0.02, 0.22 ± 0.02, 0.17 ± 0.02, and 0.24 ± 0.02, correspondingly (Table 3). Albeit not presenting statically significant changes in the roughness parameters, slightly higher values could be associated with the formation of Ag nanograins and their coalescence, as observed in Figure 2B.1.

Figure 3A.1 displays representative contact angle images of water, formamide, and diiodomethane for SS and SS-Ag surfaces. The native stainless-steel surface exhibited average contact angles of 73.17°, 70.77°, and 42.90° for water, formamide, and diiodomethane, respectively (Figure 3A.2). Silver ion implantation led to a significant decrease in the formamide contact angle to an average of 59.68° (*p* < 0.05), while the diiodomethane contact angle showed a significant increase (*p* < 0.05). Notably, the water contact angle remained largely unchanged. SFE calculations using the Owens–Wendt–Rabel–Kaelble (OWRK) approach (Figure 3A.3) revealed comparable total SFE (γtot) values between both groups.

The Ag ion release profile from the SS-Ag surface in artificial saliva is illustrated in Figure 3B. The release assay was conducted for 21 days to assess the long-term release kinetics. The concentration was of 44 ± 3.0 ppb during the first 3 days and then markedly increased to 99 ± 6.0 ppb by day 7. From this timepoint, the release reached a slower and more sustained profile up to 21 days in which a total release of 119 ± 5.0 ppb was achieved. This release pattern is clinically relevant, as localized Ag ion concentrations around the bracket are expected to exert a bactericidal effect through direct contact with bacterial cells, thereby preventing the biofilm formation.

In the case of mechanical properties, measurements were performed under low loads to ensure that the indentation depth remained within the Ag-implanted layer (~10 nm), thereby characterizing the mechanical properties of the modified region specifically. The results demonstrated that the surface enrichment with Ag ions was associated with a significantly higher hardness (3.34 ± 0.14, *p* < 0.05) compared to the unmodified stainless-steel substrate (Table 4). The static (µs) and dynamic (µd) friction coefficients were evaluated under dry and artificial saliva-lubricated conditions and results are summarized in Table 5. SS-Ag exhibited significantly lower friction coefficients than SS brackets under both conditions. In all groups, dynamic friction was lower than static friction, while artificial saliva further reduced these values. These findings indicate that the tribological performance of the brackets–wire interface is enhanced due to the synergistic effect of the salivary lubrication on Ag-implanted substrates.

### 3.2. In Vitro Biocompatibility

The cytocompatibility of SS and SS-Ag was evaluated indirectly using extracts collected after 1, 3, 7, 14, and 21 days of sample immersion in supplemented DMEM or supplemented FGM. As depicted in Figure 4A.1,A.2, the cell viability of both HGF and HFF cells remained close to or above 100% of the control, with no significant differences between SS and SS-Ag. From the results, it can be concluded that the Ag release described in Section 3.1 does not induce cytotoxicity over the 21-day evaluation time.

### 3.3. In Vitro Protein Adsorption of Salivary Proteins for Acquired Pellicle Formation Assessment

Given the selective nature of the salivary protein adsorption onto orthodontic brackets, this study aimed to elucidate the adhesion of two major constituents of the acquired pellicle onto orthodontic brackets that later mediate the attachment of primary bacteria colonizers such as *S. gordonii*. For this purpose, α-amylase and mucin adsorption was evaluated independently. While there were no statistically significant differences between the adsorbed protein onto SS-Ag when compared to SS, there was a lower variability of α-amylase adhesion when Ag ions were implanted onto stainless-steel surfaces (Figure 4B.1–B.3). Interestingly, mucin adsorption remained relatively low, accounting for approximately 2% of the total protein available in the solution. Conversely, around 46% of total amylase was absorbed onto the surface of both SS and SS-Ag. These results suggest that the modifications of the physicochemical characteristics of the stainless steel by the Ag implantation would not hinder the formation of the acquired salivary pellicle.

Water contact angle measurements further demonstrated the influence of the adhered salivary proteins on the surface wettability (Figure 4C.1–C.3). Adsorption of either mucin or α-amylase significantly reduced the contact angle on both substrates. Although SS-Ag tended to exhibit lower contact angles than SS following protein adsorption, both surfaces displayed the same overall trend, indicating that salivary proteins increased surface hydrophilicity regardless of the substrate.

Protein adsorption similarly had an effect on the brackets surface topography as depicted in Figure 5. Three-dimensional topographical maps revealed that mucin adsorption led to a modest change in the surface features, while α-amylase produced more significant changes, particularly on SS-Ag. In detail, the maximum peak height (Sp), maximum valley depth (Sv), and total height (St) were increased to 4.87 ± 2.35 µm, 11.34 ± 4.88 µm, and 16.21 ± 2.86 µm on SS surfaces, whereas these parameters increased to 7.37 ± 1.95 µm, 16.63 ± 9.27 µm, and 23.99 ± 10.77 µm, respectively, on SS-Ag substrates (Table 6). In contrast, the average roughness parameters Sa and Sq remained unchanged for both substrates following protein adsorption. Altogether, these findings suggest that protein adsorption generated localized topographical features that could facilitate bacterial attachment.

The difference between roughness values obtained from the group with no protein adsorption in this assay (Table 6) and the values reported in Section 3.1 (Table 3) is because in the protein adsorption assays flat samples were considered instead of the Bios stainless-steel brackets analyzed by SEM, EDS, and optical interferometry.

### 3.4. In Vitro Antibacterial Activity

The antibacterial performance of SS and SS-Ag^+^ brackets were assessed by CFU counting (Figure 6A) and resazurin-based metabolic activity assay (Figure 6B) against seven oral bacterial species (*S. mutans*, *S. aureus*, *L. salivarius*, *L. acidophilus*, *P. gingivalis*, *E. faecalis*, and *S. gordonii*). Significant reductions in viable bacterial counts (*p* < 0.05) were observed for *S. aureus*, *L. salivarius*, *L. acidophilus*, *P. gingivalis*, and *E. faecalis* on SS-Ag^+^ surfaces compared to SS controls. These results were corroborated by the metabolic activity assay, which showed significantly lower resazurin reduction percentages for the same species on implanted surfaces. In contrast, no statistically significant differences were detected for *S. mutans* or *S. gordonii*, suggesting a species-dependent antibacterial response. In Table 7 quantitative data for bacterial reductions are shown with *p* values.

## 4. Discussion

The present study demonstrated that Ag ions implantation provides a multifunctional surface modification for stainless-steel orthodontic brackets by improving the tribological behavior, providing selective antibacterial activity, and maintaining cytocompatibility.

As orthodontic brackets are constantly exposed to saliva, the selective adsorption of salivary proteins leads to the formation of an acquired pellicle that promotes subsequent biofilm development. Beyond surface roughness, surface wettability and surface free energy (SFE) play a crucial role in bacterial adhesion. It has been demonstrated that Ag implantation significantly increased the polar component (γp) while correspondingly decreasing the dispersive component (γd). These results indicate that silver implantation alters surface polarity and chemical functionality rather than macro-hydrophilicity per se, a shift that could significantly modulate protein adsorption dynamics, acquired pellicle formation, and subsequent bacterial colonization.

Silver-based surface modifications have been extensively investigated as potential strategies to provide antibacterial properties to dental materials based on the already elucidated mechanisms of action of silver such as the disruption of bacterial membranes, interference with metabolic pathways, generation of oxidative stress, interaction with bacterial DNA, and reduced bacterial adhesion [28,29,30,31]. These studies have included different approaches such as coating orthodontic brackets or arch wires with silver nanoparticles [16]. As an example, silver nanoparticles (Ag-NPs), electrodeposited on arch wires have shown a bacterial reduction above 90% of the control [32]. Ag-NPs of 8.1 nm were capable of hindering *S. mutans* adhesion on brackets in a 98.8% [33]. Other silver formulations like the in situ synthesis of NanoAg within PMMA (NanoAg-IS-BOA) has shown higher reduction in dental biofilm early colonizers (74.1% for *S. mutans*; 79.9% for *S. sobrinus*; 2–70-fold-change across all species) compared to NanoAg incorporated by conventional mixing (NanoAg-I-BOA; 20.1% and 25.4% respectively) [34]. However, these coatings are susceptible to progressive wear and detachment that is associated with the release of debris into the oral cavity.

The controlled, sub-toxic silver release observed (119 ± 5 ppb) reflects an intentional trade-off between long-term cytocompatibility and antimicrobial potency. While this trace ion release guarantees absolute non-cytotoxicity toward human fibroblasts, it may represent a sub-inhibitory concentration for specific early-colonizing oral streptococci. *Streptococcus mutans* and *Streptococcus gordonii* possess thick peptidoglycan cell walls and robust exopolysaccharide (EPS) extracellular matrices that impede the penetration of low-dose silver ions. Consequently, antibacterial efficacy on SS-Ag surfaces is predominantly driven by a direct contact-killing mechanism rather than a high-dose release effect. While this localized action successfully eradicated or significantly reduced bacterial loads of *S. aureus*, *Lactobacillus* spp., *P. gingivalis*, and *E. faecalis*, higher local ionic thresholds or synergistic approaches may be required to achieve complete inhibition against resilient streptococcal biofilms

Unlike conventional coatings with silver nanoparticles (Ag-NPs) [35,36,37,38], the near-surface layer of orthodontic brackets in this study were enriched with Ag ions via plasma-based implantation. This represented an advantage as it eliminated the risk of delamination or nanoparticle release due to frictional loading, cyclic stress or pH fluctuations to which orthodontic brackets are exposed during clinical use. In addition, the metal ion implantation did not produce significant modifications of the surface morphology. SS-Ag surfaces also exhibit increased surface hardness, which might contribute to improve the wear resistance and reduce the metallic debris generation during the arch wire sliding in the bracket. Nevertheless, the ion enrichment via plasma treatment was responsible for a slight increase in the polar component that led to a slight increase in the surface free energy and hydrophilicity. These surface changes are relevant because bacterial adhesion is influenced by the physicochemical interaction between microorganisms and the material surface.

The clinical relevance of the variation in the mechanical properties of SS-Ag surfaces has already been highlighted in previous studies that evaluated the effect of antibacterial coatings on the mechanical and tribological behavior or orthodontic brackets. For example, Ghasemi et al. reported that nano-titanium dioxide coatings significantly increased frictional forces (1.52–1.57 N) compared with uncoated brackets (0.55 N), whereas nano-silver coatings did not produce statistically a significant increase in friction (0.77–0.82 N) [30]. Similarly, Tawakal et al. demonstrated that coatings with Ag-NPs and Ag/chitosan NPs significantly increased the static friction of metallic brackets but no effect was observed when applied on ceramic brackets [39,40,41].

The increased wettability of SS-Ag surfaces also has a clinical relevance from the standpoint of the mechanical properties of the materials as it is expected to promote the formation of a stable salivary film on the bracket, thereby reducing the direct metal-to-metal contact when the arch wire is placed. This mechanism will be consistent with the lower static and dynamic friction coefficients observed in this study for the SS-Ag samples (Figure 7 illustrates the proposed mechanism for the reduction in friction coefficients in the presence of saliva).

Ag ions release pattern also differed from previous reports. Compared with the study by Ryu et al. [42], the present work showed a lower but sustained Ag release profile. The SS-Ag^+^ surface released 44 ± 3 ppb during the first 3 days, increasing to 99 ± 6 ppb by day 7 and reaching a cumulative concentration of 119 ± 5 ppb after 21 days. In contrast, Ryu et al. reported Ag concentrations of 1.94–2.04 ppm (1940–2040 ppb) after 8 days of immersion in PBS for Ag–Pt-coated stainless-steel orthodontic brackets, regardless of the percentage of Ag coating (93–100%), whereas no Ag release was detected from the uncoated control. These differences can be attributed to the chosen deposition method and to the variances in the immersion medium (PBS and artificial saliva). Despite these quantitative differences, both studies demonstrated sustained Ag release capable of providing antibacterial activity against oral pathogens. Notably, the lower Ag release observed in the present study may represent an advantage for long-term orthodontic applications.

Albeit triggering some changes in the roughness, wettability and SFE of the treated stainless-steel substrates, the Ag implantation did not affect the adsorption of mucin or α-amylase. These results are in agreement with previous reports that showed that silver-containing surfaces do not hinder salivary protein adsorption or the biological functionality of the attached salivary proteins. Once adsorbed onto the surface, both mucin and amylase increased the wettability. Considering that the bacterial colonization of the orthodontic brackets is mediated primarily by the acquired salivary pellicle, it can be concluded that the Ag superficial incorporation does not prevent the salivary pellicle formation. Consequently, the antibacterial effects observed in this study are more likely related to the bacterial contact with the release of Ag ions than to the reduction in salivary protein binding sites. In this regard, the antibacterial evaluation revealed a species-dependent response to silver ion implantation. Significant reductions in bacterial viability were observed for *L. salivarius*, *L. acidophilus*, *P. gingivalis*, *E. faecalis*, and *S. aureus*. The inhibition of *P. gingivalis* is particularly relevant because this pathogen is strongly associated with periodontal inflammation and peri-implant diseases. Similarly, the reduction in *E. faecalis* viability is clinically important as it is reported as one of the primary etiological agents of persistent endodontic infections and is recognized as one of the most challenging microorganisms to eradicate from root canal systems [43,44].

Although *S. aureus* is not a primary constituent of the commensal oral microbiota, its inclusion in the present antibacterial evaluation was clinically justified. Several studies have reported the recovery of *S. aureus* from orthodontic brackets both as received from manufacturers and following clinical exposure, suggesting that appliance surfaces can act as reservoirs for this opportunistic pathogen [45]. Strains isolated from orthodontic appliances have demonstrated high adhesion potential to abiotic surfaces and the capacity to persist within multispecies biofilms [46], raising concerns regarding cross-contamination in the clinical setting. Furthermore, *S. aureus* carried in the oral cavity has been associated with cervicofacial infections and other opportunistic conditions when host defenses are compromised [47] and the emergence of methicillin-resistant strains (MRSA) in the oral environment represents an additional public health concern [48,49]. In the present study, Ag ions implantation produced a significant reduction in *S. aureus* viability on bracket surfaces, a finding that further supports the potential of this surface modification to reduce the risk of cross-contamination and opportunistic infection in the orthodontic clinical environment.

In contrast, no statically significant reduction was observed for *S. mutans* and *S. gordonii*. These findings indicate that the antibacterial efficacy of Ag ions is not solely determined by the number of ions being released from the surface but also by the biological characteristics of the bacteria such as the cell wall structure, membrane composition, surface charge, metabolic activity or the stress-response mechanisms of each bacteria strain.

Furthermore, the increased hydrophilicity and polar component of the surface free energy induced by the Ag ion implantation may influence differently the bacterial adhesion depending on the bacterial strains. In detail, *S. gordonii* is an early colonizer that adheres efficiently to amylase and mucin through specific adhesin-receptor interactions, whereas *S. mutans* mostly relies on glucan-mediated attachment that in this study was promoted due to the presence of 5% sucrose that acted as precursor of glycans in simulated cariogenic conditions [50,51]. Altogether, both strains were able to synthesize extracellular polysaccharides that reinforced the bacterial attachment and biofilm formation which would partially explain why the Ag ion release was not sufficient to reduce colonization by these cariogenic *streptococci*. Hence, the final antibacterial performance is likely governed by the combined effects of silver activity and the bacteria-surface interactions [52,53]. In this scenario, Ag ions implantation alone may not be sufficient to effectively suppress the bacterial species most directly associated with dental caries.

Based on these results, it is proposed that future research should be focused on understanding how the variations in bacteria membrane composition, surface charge, and metabolic activity can influence the silver bactericidal effect. For this purpose, advanced microscopic and molecular analyses could help determine whether Ag ions penetrate the bacterial cells and to later identify the intracellular pathways affected by their intracellular presence.

Regarding the biocompatibility of the modified materials, Ag ions release assays resulted in free ion concentrations in a parts-per-billion range, indicating a very low level of ion release that were accompanied by excellent cell viability percentages when evaluated in two types of fibroblasts cell lines. The obtained values are above the 70% threshold of cell viability indicated in the commonly accepted ISO 10993-5 [22] for biomedical materials evaluation. As concerns regarding the long-term safety of silver-based antibacterial materials have been recently raised [54], the cytocompatibility assays results highlight the benefits of dental materials modifications using plasma-based techniques.

The increase in surface hardness induced by Ag ion implantation plays a crucial role in improving tribological behavior. Higher surface hardness resists localized plastic deformation of the bracket slot during engagement with the arch wire. By mitigating micro-scale plowing and surface damage, the overall friction coefficient is effectively reduced, thereby enhancing arch wire sliding efficiency. Taken together, the results of this study indicate that Ag ions implantation confers a multifunctional improvement to stainless-steel orthodontic brackets, simultaneously enhancing tribological performance, providing selective antibacterial activity against key periodontal and endodontic pathogens, and maintaining no detection of cytotoxicity under the tested short-term in vitro conditions. These combined properties position silver ion implantation as a clinically promising surface modification strategy for next-generation orthodontic devices.

Despite these promising findings, several limitations of the present study should be acknowledged. First, the experiments were conducted strictly in vitro and under short experimental durations, which do not fully replicate the long-term, complex biological environment of orthodontic treatment. Second, the antibacterial efficacy was evaluated using single-species cultures rather than a multispecies biofilm model. This represents a notable limitation, as oral biofilms rely on interspecies interactions that strongly modulate bacterial adhesion, matrix production, and antimicrobial resistance.

Furthermore, the present study did not incorporate long-term aging, mechanical wear, or corrosion testing, which are critical for assessing the structural stability and sustained durability of the Ag-implanted layer over extended clinical periods. Mechanistically, while surface composition was evaluated, depth profiling, silver chemical-state analysis (e.g., XPS), and the stability of the ion-implantation layer were not performed. Finally, artificial saliva was used as a simplified environment, omitting the complex salivary pellicle and protein interactions that occur in vivo. Therefore, future studies incorporating multispecies dynamic biofilms, detailed depth profiling, tribocorrosion testing, and clinical trial conditions are required to validate the long-term performance of these modified brackets.

Based on the experimental findings, both null hypotheses were rejected. Regarding the first null hypothesis, silver ion implantation demonstrated marked antibacterial efficacy against key oral pathogens (including *Lactobacillus* spp., *P. gingivalis*, *E. faecalis*, *and S. aureus*), thereby rejecting the premise of antibacterial inactivity. Regarding the second null hypothesis, rather than impairing tribological behavior, the treatment significantly enhanced sliding performance at the bracket–arch wire interface. This improvement is directly attributed to the increased surface nano-hardness and surface energy imparted by the Ag implantation, which reduced the friction coefficient under both dry and saliva-lubricated conditions. Consequently, this modified surface holds strong clinical potential to optimize orthodontic sliding mechanics and potentially reduce overall treatment duration.

## 5. Conclusions

Plasma-based Ag ion implantation provided a multifunctional stainless-steel orthodontic bracket surface characterized by and enhanced surface hardness and tribological performance while maintaining the original surface morphology, promoting a slightly higher wettability, and biocompatible treatment. As a result, the modified surface exhibited a good antibacterial activity against clinically relevant pathogens in dentistry such as *P. gingivalis*, *E. faecalis*, *L. salivarius*, and *L. acidophilus*. However, the silver-enriched surfaces did not show antibacterial efficacy against cariogenic *streptococci*, demonstrating that Ag ions release is not sufficient to control the biofilm formation of these bacteria strains. Overall, the plasma-based implantation of Ag ions described in this study represents a promising alternative to conventional coatings with Ag-NPs by providing antibacterial functionality alongside improved mechanical performance of the orthodontic brackets. Further studies using multispecies biofilm models and long-term in vivo conditions are still required to validate the performance of the modified surfaces under clinical conditions.

## Figures and Tables

**Figure 1:**
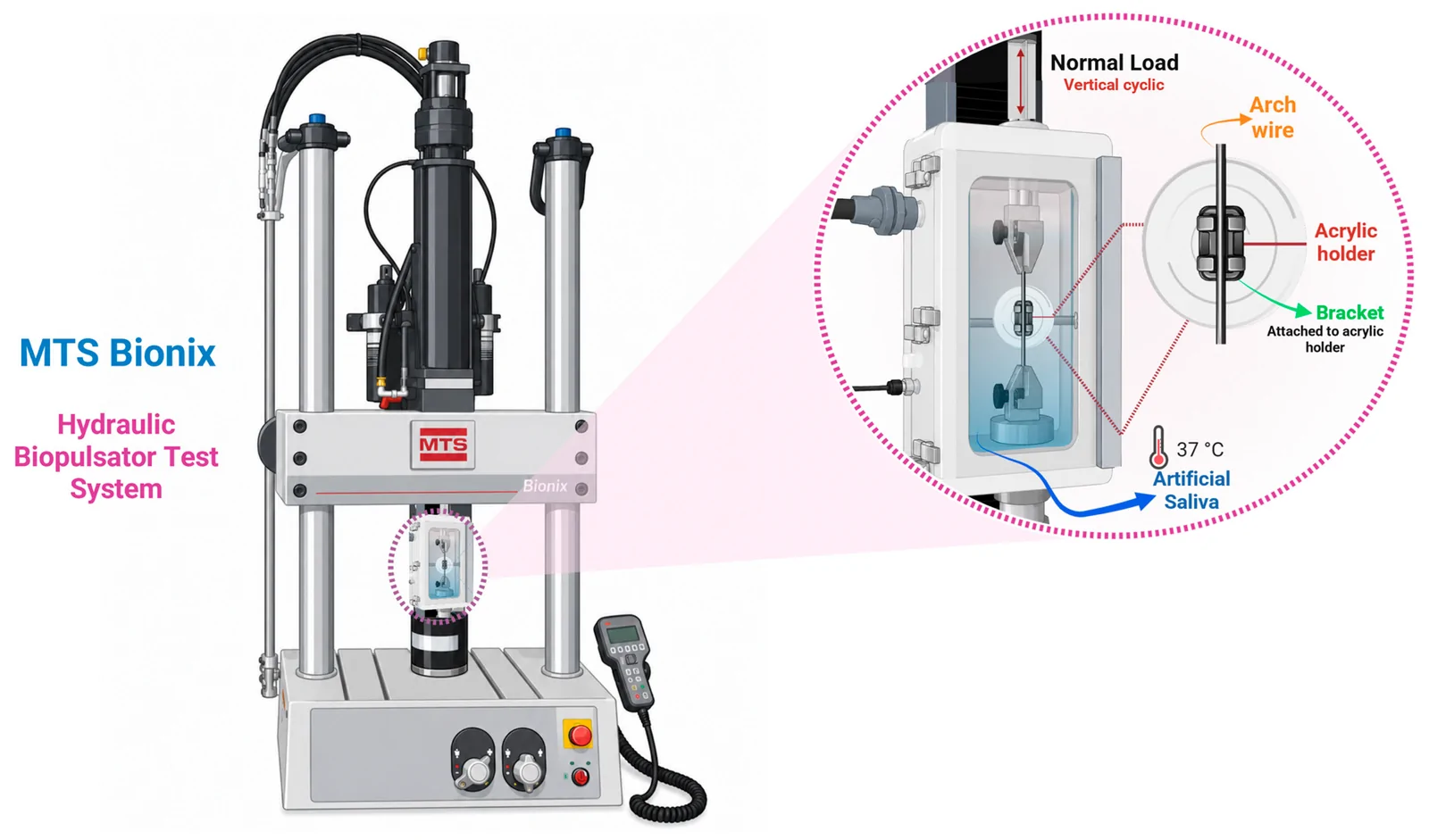
Set up for the tribological testing used to determine the static and dynamic friction coefficients at the brackets–arch wire interface under oral environment conditions: the bracket, SS or SS-Ag, was mounted on a holder and loaded with a controlled normal force, while the stainless-steel arch wire was displaced at a constant rate. As shown, samples were immersed in artificial saliva at 37 °C, simulating oral environment.

**Figure 2:**
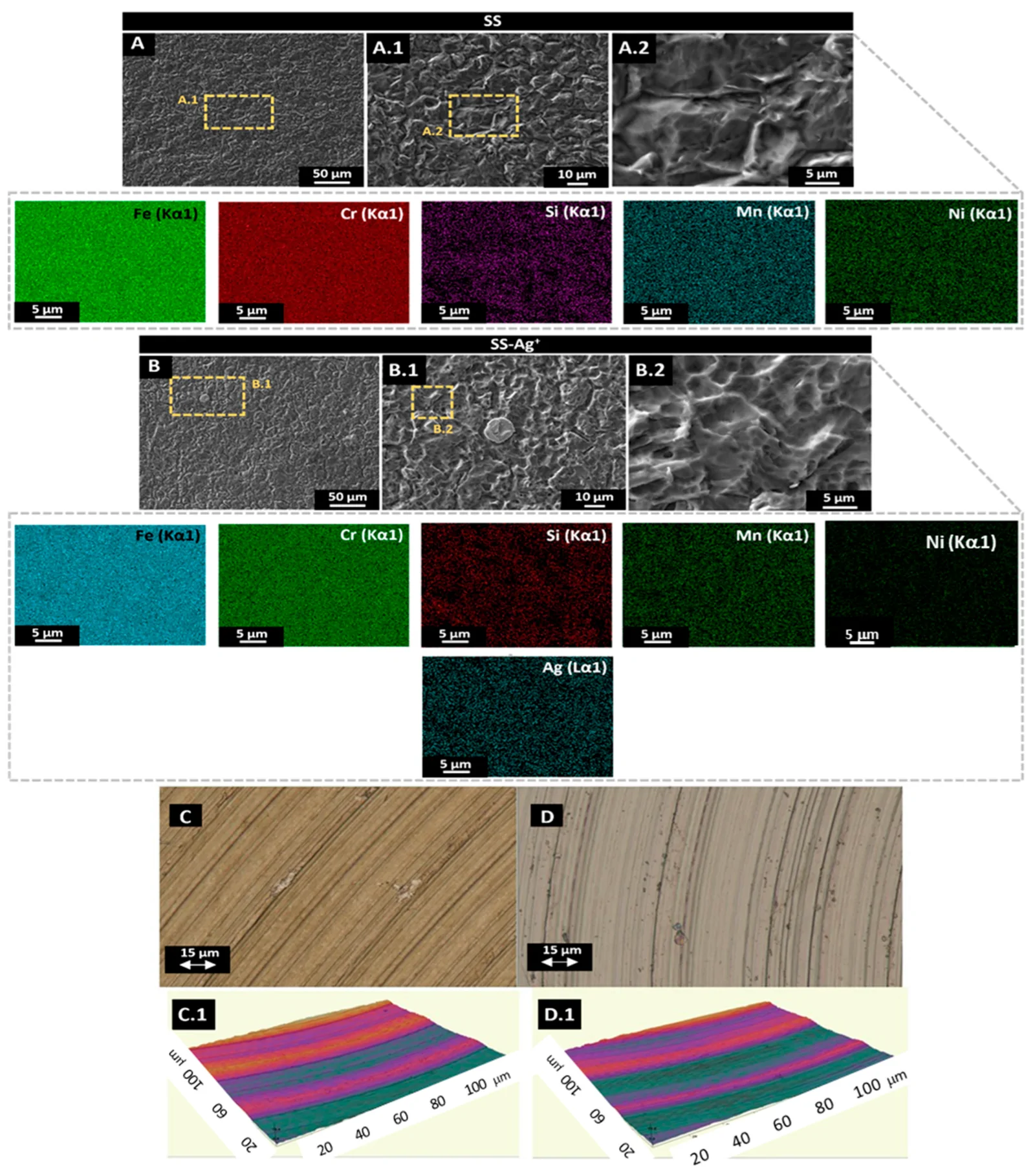
Surface characterization of unmodified stainless steel (SS) and Ag-implanted stainless steel (SS-Ag): representative SEM micrographs of SS and SS-Ag are depicted in (**A**–**A.2**) and (**B**–**B.2**), correspondingly. Higher magnification SEM images (**A.2**,**B.2**) were used for EDS mapping to confirm the elemental composition, demonstrating a homogenous distribution of Ag on the SS-Ag surface. The near-surface structural modifications induced by the ion implantation treatment did not alter the original surface topography as demonstrated by interferometry results shown in (**C**,**C.1**) for SS samples and (**D**,**D.1**) for SS-Ag.

**Figure 3:**
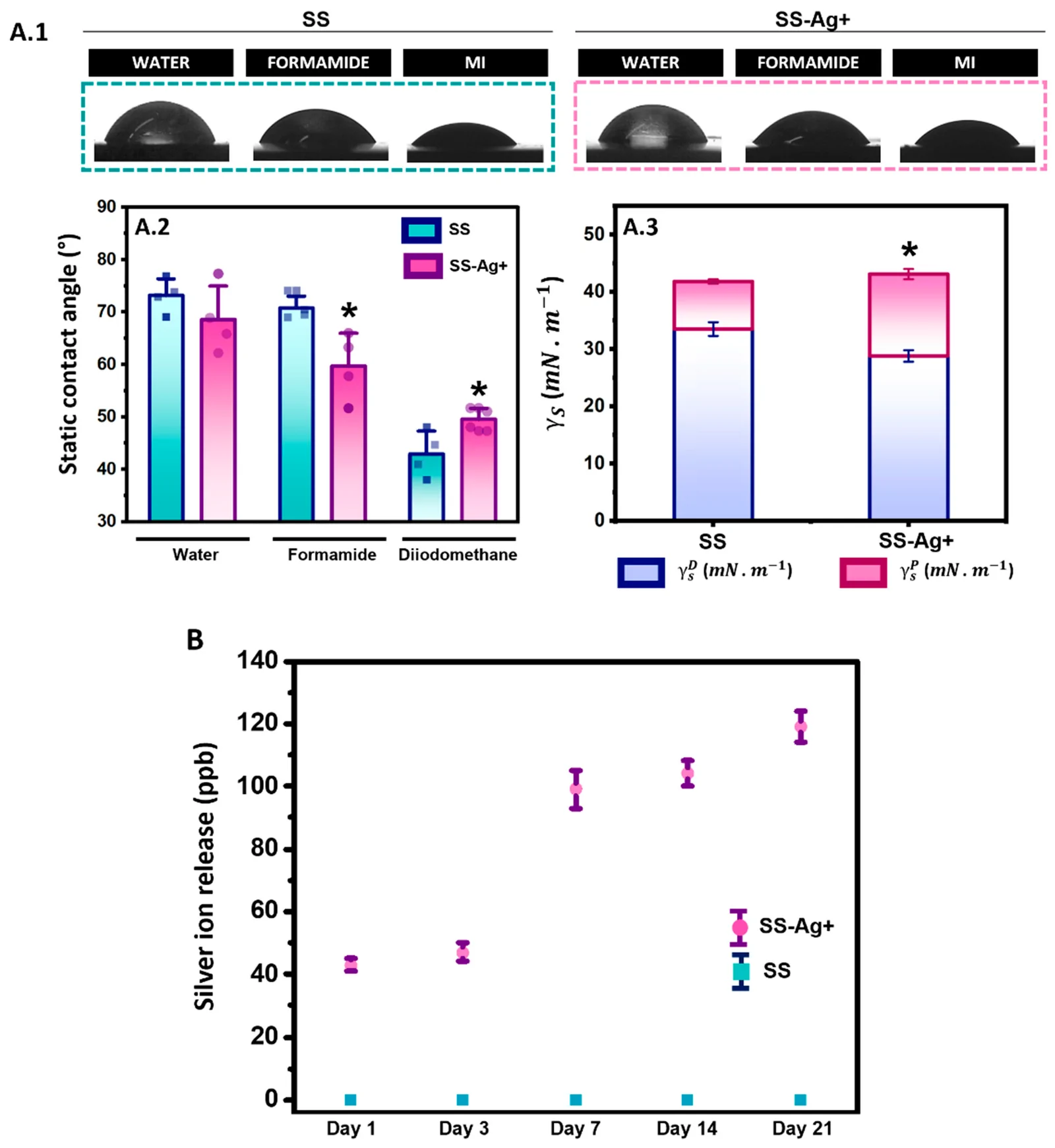
Surface wettability (representative micrograph in (**A.1**) and mean ± SD in (**A.2**)), surface free energy (SFE) in (**A.3**), and silver ion release of unmodified stainless steel (SS) and silver-ion-implanted stainless steel (SS-Ag). The asterisks indicate statistically significant differences with a *p-*value *<* 0.05 between the SS and SS-Ag brackets for each solvent and for the surface total energy. (**B**). The representative contact angle images shown in (**A.1**) were obtained with water, diiodomethane, and formamide, the same liquid probes used to calculate SFE by the Owens–Wendt–Rabel–Kaelble (OWRK) method. The ICP–MS analysis demonstrated sustained Ag release from SS–Ag brackets over 21 days in artificial saliva. Values are expressed as mean ± SD.

**Figure 4:**
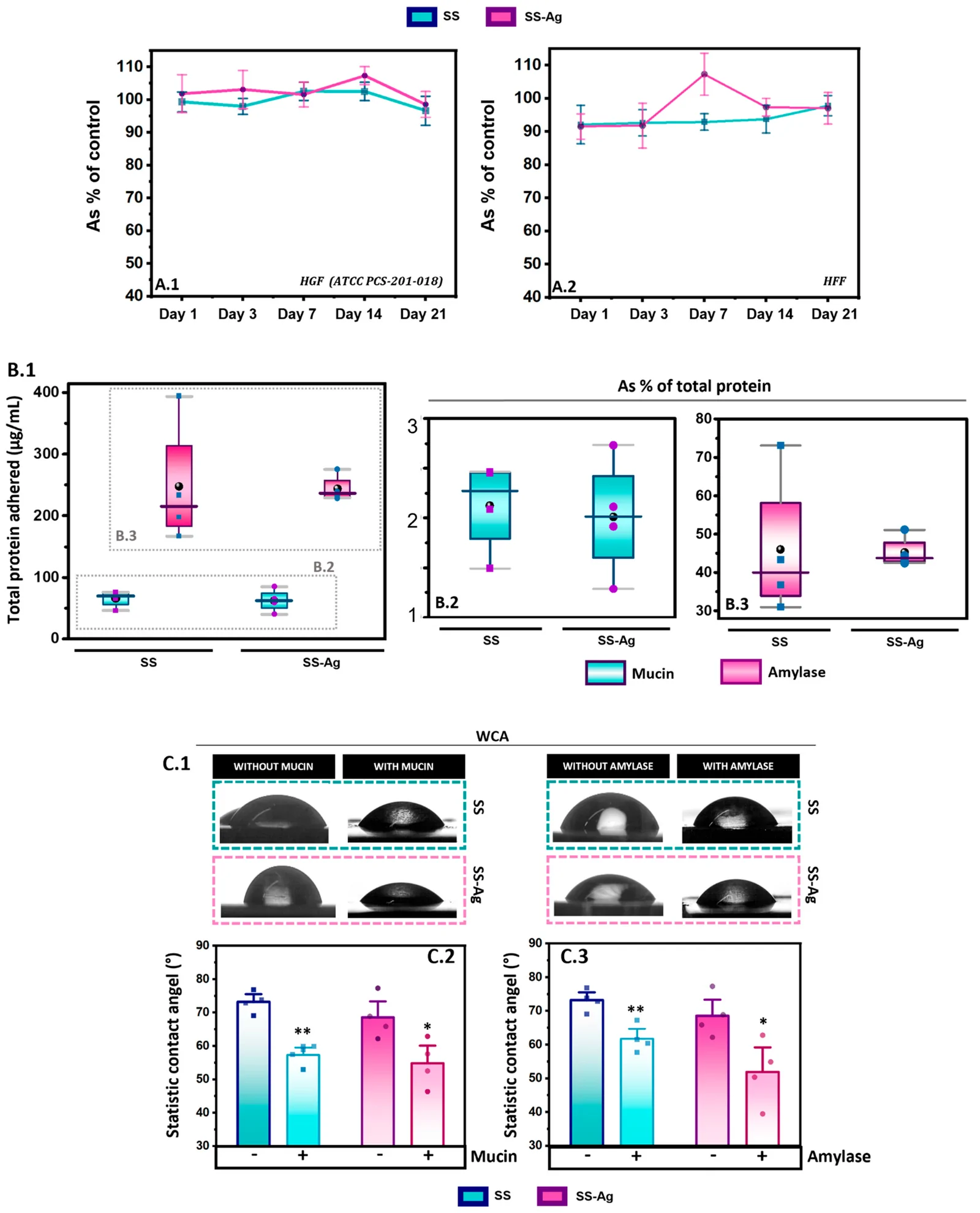
Biocompatibility, salivary protein adsorption, and surface wettability following protein adsorption of SS and SS-Ag samples. (**A.1**) Cell viability of human gingival fibroblasts (HGF) and (**A.2**) human foreskin fibroblasts (HFF) exposed to 1-, 3-, 7-, 14- or 21- extracts of SS and SS-Ag samples. Cell viability is expressed as the percentage of the control (considering that the fluoresce values obtained in this group are equal to 100%). (**B.1**) Salivary protein adsorption. Total adsorbed protein (µg/mL), (**B.2**) mucin protein adsorption, and (**B.3**) amylase adsorption, both expressed as percentage of the total protein in artificial saliva solution. In (**B.1**–**B.3**), the black circles represent the mean values, and the colored circles represent the various experimental results. (**C.1**) Surface wettability analysis by static water contact angle (WCA): representative drop images of SS and SS-Ag surfaces in the absence or presence of adsorbed mucin or amylase, (**C.2**) quantitative WCA measurements following mucin and (**C.3**) amylase. In all cases, statistical significance is indicated as *p* < 0.05 (*) and *p* < 0.01 (**) when protein treated surfaces were compared to the control (with no protein adsorption). In (**C.2**,**C.3**), the black squares represent the mean values, and the other symbols correspond to the various experimental results.

**Figure 5:**
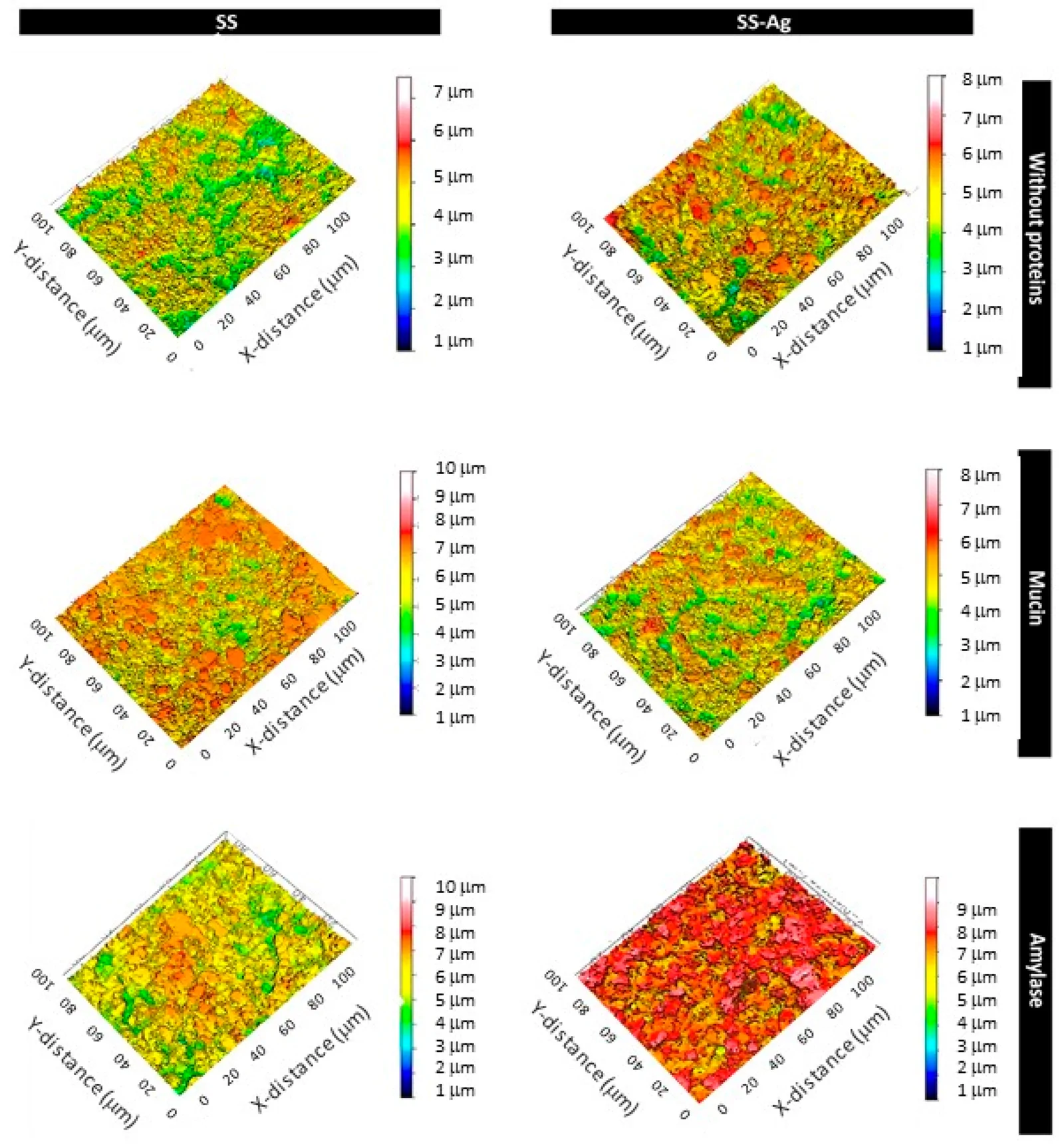
Three-dimensional surface topography of SS and SS-Ag samples before and after salivary protein adsorption. Representative 3D WLI images in the absence of proteins (without proteins) and following adsorption of mucin or amylase. Surface topography was acquired over a 100 × 100 µm^2^ scanning area.

**Figure 6:**
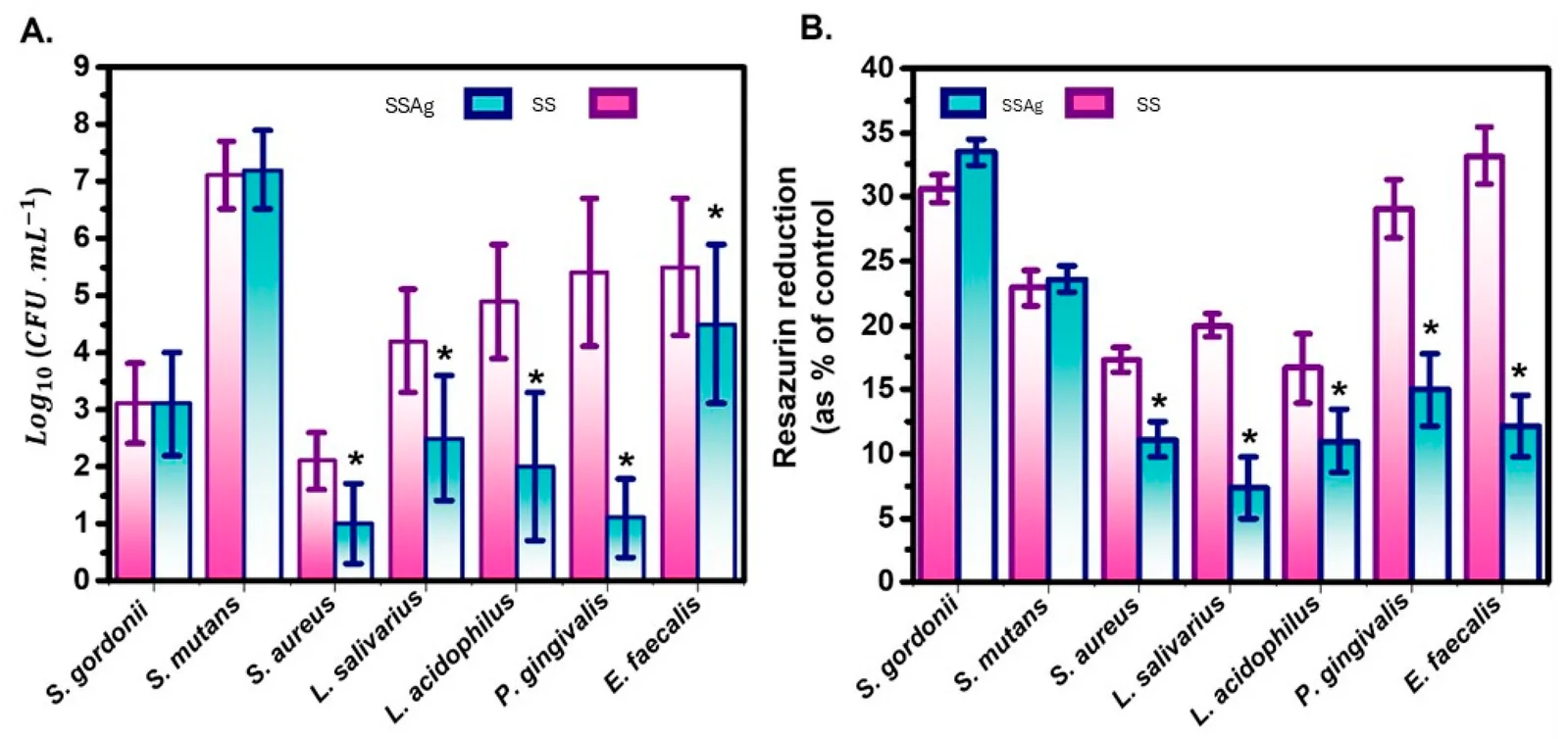
Antibacterial activity of SS and SS-Ag brackets surface against most common oral pathogens. (**A**) Viable bacterial counts expressed as log CFU. mL^−1^. (**B**) Metabolic activity of analyzed bacteria *(S. mutans*, *S. aureus*, *L. salivarius*, *L. acidophilus*, *P. gingivalis*, *E. faecalis*, *and S. gordonii*) expressed as percentage of resazurin reduction (%). Data are presented as mean ± SD. * *p* < 0.05 when SS-Ag is compared to SS.

**Figure 7:**
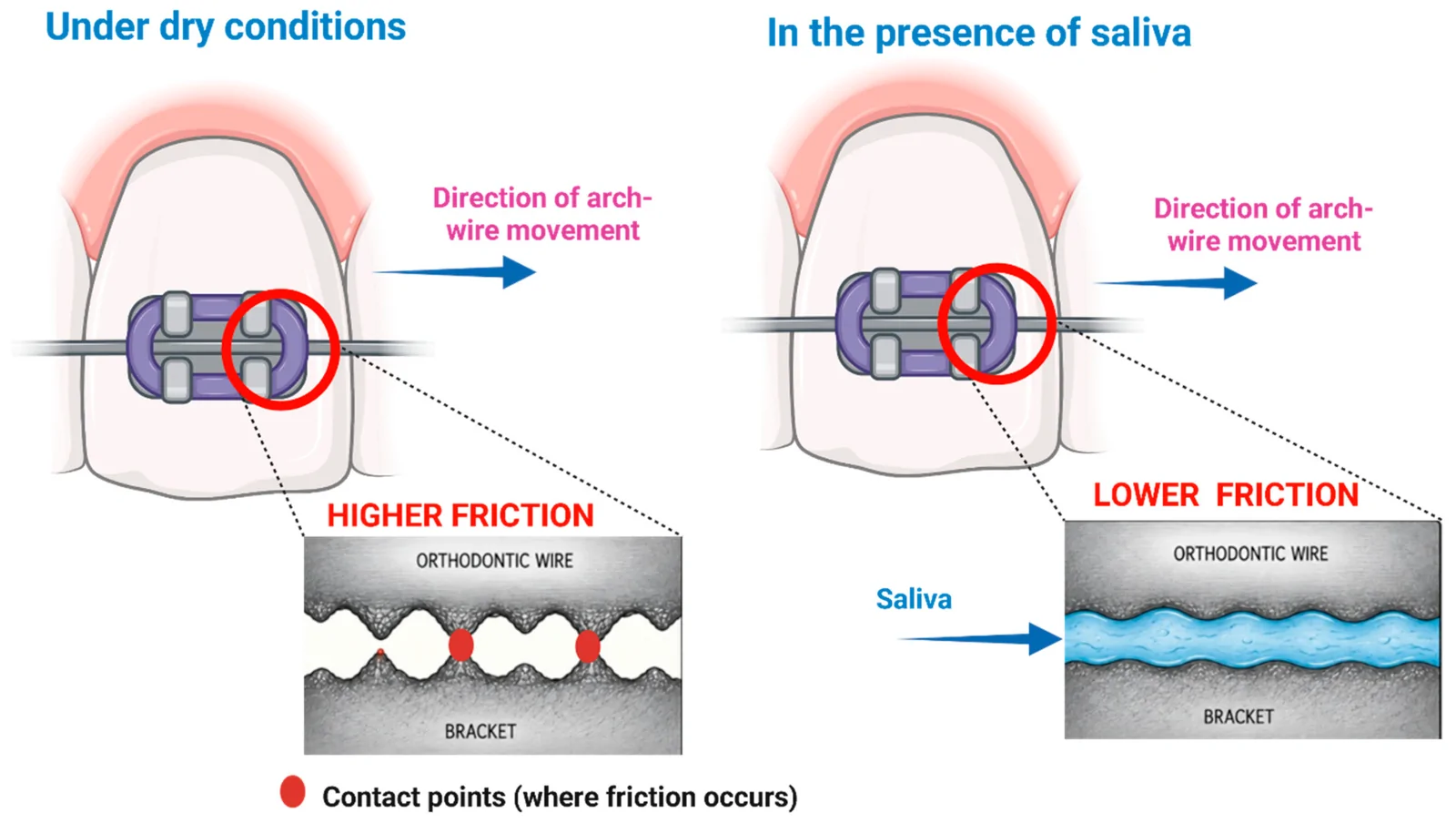
Illustration of the proposed mechanism for the friction reduction at the orthodontic bracket–arch wire interface under wet conditions (presence of saliva). In dry conditions, the direct metal to metal contact results in high frictional resistance. Conversely, saliva forms a lubricating film that reduces the number of contact points and lowers both static and dynamic friction coefficients.

**Table 1:** Composition of the artificial saliva used in this study.

Reagent	Concentration (g/dm^3^)
K_2_HPO_4_	0.22
KCl	1.19
KSCN	0.29
Na_2_HPO_4_	0.26
NaCl	0.69
NaHCO_3_	1.49
Urea	1.49
Lactic Acid	until pH = 6.8

**Table 2:** Element content (Atomic %) as determined by EDS (± SD).

Sample	Si	Cr	Mn	Fe	Ni	Ag
SS	0.41 ± 0.07	18.45 ± 0.04	1.25 ± 0.02	73.49 ± 0.06	6.42 ± 0.06	0
SS-Ag	0.37 ± 0.03	18.26 ± 0.02	1.25 ± 0.09	72.99 ± 0.29	6.22 ± 0.22	0.93 ± 0.60

**Table 3:** Surface roughness parameters of evaluated samples (± SD) and *p*-values.

Sample	*S_a_* (µm)	*p*	*S_m_* (µm)	*p*	Index Area	*p*
Arch wire	0.12 ± 0.01	0.08	0.14 ± 0.05	0.20	1.0 ± 0.04	0.09
SS	0.16 ± 0.02	0.12	0.22 ± 0.02	0.22	1.09 ± 0.03	0.11
SS-Ag	0.17 ± 0.02	0.17	0.24 ± 0.02	0.24	1.10 ± 0.02	0.12

**Table 4:** Surface hardness (GPa) of arch wires, SS and SS-Ag brackets (± SD). Asterisk indicates the statistical difference significance with *p* < 0.05.

Sample	*H* (GPa)
Arch wire	2.90 ± 0.29
SS	2.72 ± 0.23
SS-Ag	3.34 ± 0.14 *

**Table 5:** Static (μ_s_) and dynamic (μ_d_) friction coefficients for SS and SS-Ag samples under dry and artificial saliva-lubricated conditions (mean ± SD). Asterisk (*) indicates statistically significant differences (*p* < 0.05).

Sample	Dry		Artificial Saliva	
	μ_s_	μ_d_	μ_s_	μ_d_
SS	0.21 ± 0.03	0.18 ± 0.07	0.18 ± 0.07	0.11 ± 0.07
SS-Ag	0.12 ± 0.04 *	0.06 ± 0.04 *	0.08 ± 0.02 *	0.05 ± 0.04 *
*p*-value	0.023	0.028	0.032	0.030

**Table 6:** Roughness values of samples before and after protein adsorption (W/O: without protein).

Roughness Parameters (µm)	SS				SS-Ag	
	W/O (*n =* 3)	Mucin (*n =* 3)	Amylase (*n =* 3)	W/O (*n =* 3)	Mucin (*n =* 3)	Amylase (*n =* 3)
S_p_	3.99 ± 1.44	7.27 ± 3.65	4.87 ± 2.35	3.54 ± 0.58	4.84 ± 0.27	7.37 ± 1.95
S_v_	6.58 ± 2.72	9.34 ± 5.39	11.34 ± 4.88	7.81 ± 4.08	8.72 ± 4.46	16.63 ± 9.27
S_t_	10.57 ± 3.63	16.60 ± 8.70	16.21 ± 2.86	11.35 ± 4.60	13.56 ± 4.60	23.99 ± 10.77
S_a_	0.52 ± 0.07	0.71 ± 0.54	0.54 ± 0.03	0.55 ± 0.03	0.53 ± 0.02	0.54 ± 0.03
S_q_	0.65 ± 0.08	0.88 ± 0.60	0.69 ± 0.04	0.69 ± 0.04	0.66 ± 0.01	0.69 ± 0.04

*S_p_*: Maximum peak height, *S_v_*: Maximum valley depth, *S_t_*: Maximum height of the surface, *S_a_*: arithmetical mean height, *S_q_*: Root mean square height.

**Table 7:** Quantitative reduction in CFU for the different bacteria strains and *p-*values.

Bacteria Strain	Reduction (%)	*p-*Value
*S. gordonii*	2.0	0.881
*S. mutans*	0.1	0.901
*S. aureus*	62.3	0.031
*L. salivarius*	45.6	0.025
*L. acidophilus*	72.2	0.030
*P. gingivalis*	92.7	0.012
*E. faecalis*	38.2	0.040

## Data Availability

The authors can provide details of the research requirements by letter and comments under needs.
